# Madness Decolonized?: Madness as Transnational Identity in Gail Hornstein’s *Agnes’s Jacket*

**DOI:** 10.1007/s10912-017-9434-8

**Published:** 2017-02-13

**Authors:** Gavin Miller

**Affiliations:** Medical Humanities Research Centre, University of Glasgow, Glasgow, UK

**Keywords:** Gail Hornstein, Mad identity, Voice hearing, Diaspora, Nationalism

## Abstract

The US psychologist Gail Hornstein’s monograph, *Agnes’s Jacket: A Psychologist’s Search for the Meanings of Madness* (2009), is an important intervention in the identity politics of the mad movement. Hornstein offers a resignified vision of mad identity that embroiders the central trope of an “anti-colonial” struggle to reclaim the experiential world “colonized” by psychiatry. A series of literal and figurative appeals makes recourse to the inner world and (corresponding) cultural world of the mad as well as to the ethno-symbolic cultural materials of dormant nationhood. This rhetoric is augmented by a model in which the mad comprise a diaspora without an origin, coalescing into a single transnational community. The mad are also depicted as persons displaced from their metaphorical homeland, the “inner” world “colonized” by the psychiatric regime. There are a number of difficulties with Hornstein’s rhetoric, however. Her “ethnicity-and-rights” response to the oppression of the mad is symptomatic of Western parochialism, while her proposed transmutation of putative psychopathology from limit upon identity to parameter of successful identity is open to contestation. Moreover, unless one accepts Hornstein’s porous vision of mad identity, her self-ascribed insider status in relation to the mad community may present a problematic “re-colonization” of mad experience.

Gail Hornstein is a US psychologist known for her earlier work on the psychiatrist, Frieda Fromm-Reichmann (Hornstein 2000), and her regularly updated *Bibliography of First-Person Accounts of Madness* (Hornstein 2011). Her monograph, *Agnes’s Jacket: A Psychologist’s Search for the Meanings of Madness,* was first published in the US in 2009 and then in the UK in 2012. It represents the fruits of Hornstein’s research on the psychiatric survivor movement conducted via fieldwork with the Freedom Center in Massachusetts and the Hearing Voices Network in the UK. The book takes its title from a material artefact central to the narrative, the embroidered jacket of Agnes Richter (1844-1916), a German seamstress detained in various mental health institutions from 1893 until her death. During her various periods of incarceration, Richter took to embroidering her garments with written text. The jacket, the only surviving of her embroidered garments, is now preserved in the Prinzhorn Collection in Heidelberg, alongside thousands of other artworks by asylum patients. It is covered in densely-packed cryptic phrases and sentences that seemingly allude to Richter’s life and situation, leading Hornstein to conclude that “[l]ike a coded document or a hieroglyph before the discovery of the Rosetta Stone, Agnes Richter’s jacket remains a tantalizing clue to an unknown world” (2012, x).

Hornstein’s text has been well-received across a number of disciplines and has crossed over into wider public consciousness. Since its initial publication in 2009, there have been broadly favourable reviews in specialist periodicals such as *Journal of Psychiatric and Mental Health Nursing* (Burridge 2013), *Disability and Society* (Sapey 2011), and *Journal of the American Academy of Child and Adolescent Psychiatry* (Sokoloff 2011), complemented by positive coverage in well-known newspapers such as *The Washington Times* (2009). The ongoing marketing of *Agnes’s Jacket* in later editions exploits its crossover appeal. In the UK edition (2012), for instance, the endorsements on the outside back cover include voices from the survivor movement (Jacqui Dillon of the Hearing Voices Network) and clinical psychology (Mary Boyle) but extend also to the feminist public intellectuals, Barbara Ehrenreich and Susie Orbach – the latter well-known as the author of the classic manifesto, *Fat is a Feminist Issue* (1978). Popular awareness of *Agnes’s Jacket* has been further heightened by press coverage of its cultural impact on creative arts: the text inspired a musical composition and consequent press coverage in the form of a song cycle, *Hearing Voices* (2012), by the composer Jocelyn Pook (2014), since followed by her *Anxiety Fanfare and Variations for Voices* (2014).

A psychiatric or otherwise clinical critique of *Agnes’s Jacket* might focus on Hornstein’s claims about the aetiology, ontology, and therapeutics of mental illness. Terry Burridge, for instance, is favourably disposed toward the text but finds himself “irritated by the theme in *Agnes’s Jacket* that all psychiatrists were unfeeling bullies who only wanted to pump their long suffering patients full of drugs” (2013, e4). Such an approach tends to bypass, however, interpretation and critique of Hornstein’s rallying call for the assertion by the mad^1^ of a distinct culture and identity. Although there is a strong discursive thrust to *Agnes’s Jacket*, and substantial scholarly apparatus such as endnotes and further reading, Hornstein’s book is primarily a work of advocacy for the mad movement. It incorporates a travelogue of her ethnographic fieldwork and her growing affinity with the mad as well as numerous personally-inflected interpolations, such as her encounter with Agnes’s jacket itself in a private viewing (2012, 247-249). In a retrospective attribution of political intention, Hornstein presents Agnes’s jacket as the beginning of an unfinished project that finds continuation in the contemporary mad movement: “Agnes Richter had to stitch her words into her clothes; she had no other way of communicating her ideas to the world. Today, patients have blogs, Web sites, and hundreds of local support and advocacy groups” (xxi). Hornstein’s textual intervention in this social and cultural movement has a peculiar, and somewhat contentious, character. Her text provides a bold, almost flamboyant, elaboration into madness identity of a central discursive trope: namely, the postmodern “decolonization” of mad experience through illness narrative. The aim of this article is to expound and critically discuss Hornstein’s model of the mad movement in *Agnes’s Jacket* as a diasporic nation, one slowly emerging from dormission under psychiatric “colonization” and potentially opening its borders to new citizens from outside the survivor movement.

## Decolonizing madness

In order to appreciate fully Hornstein’s distinctive intervention in mad identity politics, it is necessary to delineate some of the characteristics, and complexity, of the contemporary mad movement. As Robert Menzies et al. explain, the mad movement maintains “active lobbying, awareness, support, and mutual aid campaigns” (2013, 9) and is currently exemplified (in the Anglophone West at any rate) by

[o]rganizations like MindFreedom International (MFI), the World Network of Users and Survivors of Psychiatry (WNUSP), the European Network of (Ex)Users and Survivors of Psychiatry (ENUSP), Mad Pride UK, PsychRights, the Hearing Voices Network (HVN), the National Self Harm Network (NSHN), the Antipsychiatry Coalition, the Coalition Against Psychiatric Assault (CAPA), the National Association for Rights Protection and Advocacy (NARPA), and the International Network Toward Alternatives and Recovery (INTAR). (9)

Menzies et al. recognize “the very real differences in identities, political values, and forms of expression that mark (and sometimes set apart) the many strands of antipsychiatry, critical psychiatry, mental patients’ liberation, psychiatric survivor activism, and Mad pride” (8). They note, nonetheless, a shared vocabulary in which the words “mad” and “madness” are resignified as positive umbrella terms: “Once a reviled term that signalled the worst kinds of bigotry and abuse, madness has come to represent a critical alternative to ‘mental illness’ or ‘disorder’ as a way of naming and responding to emotional, spiritual, and neuro- diversity” (10). Consequently,

in recent years, “mad” has flooded back into the language of public culture, and into the work of critical activists and scholars worldwide. […] to take up “madness” is an expressly political act. […] madness talk and text invert the language of oppression, reclaiming disparaged identities and restoring dignity and pride to difference. (10)

Summer Schrader et al. present a similar picture in which “[v]arious groups and individuals […] have moved beyond treatment-centered activism to articulate a broader culture of madness” (2013, 62). The authors acknowledge “the heterogeneity of perspectives within the mad movement” (62) but argue that “celebrations of a shared mad culture, like the reclamation of terms such as ‘craziness’ and ‘lunacy,’ have helped solidify madness as a culturally meaningful and active sociopolitical minority identity” (62). The result is a pragmatic alliance loosely unified by a common effort to “(1) more rigorously distinguish distress from unusual mental states and (2) re-center the focus of treatment and intervention on the largely psychosocial factors that contribute to distress rather than the unusual experiences (such as voice hearing) in question” (63).

To situate *Agnes’s Jacket* within the mad movement, one must, of course, acknowledge the latter’s heterogeneity of perspectives. Although the common aims identified by Schrader et al. are clearly discernible, the ideological and practical underpinnings vary significantly as do the consequent affinities with related identities, such as the disabled persons’ movement. The theory and practice of madness may involve such diverse perspectives as: civil rights (e.g. Minkowitz 2014); anti-psychiatric abolitionism (e.g. Burstow 2014); feminism (e.g. Diamond 2014); trades unionism (e.g. McKeown et al. 2014); revolutionary politics (e.g. Burstow and LeFrancois 2014); post-colonial indigenism (e.g. Tam 2013); disability rights (e.g. Beresford and Menzies 2014); neurodiversity (e.g. McWade et al. 2015); and contemporary spirituality (e.g. Farber 2012) – and no doubt others as well. This dynamic heterogeneity makes for a commensurate diversity in models of affiliation within the mad movement. Seth Farber, for instance, sees himself as creating a spiritual community in which “the intertwined threads of madness, creativity, and collaboration can inspire hope and transformation in an oppressive and damaged world” (2012, 7). His community, though, is specifically for those who take pride in their “mad gifts,” in “having experienced altered states of consciousness, so-called ‘psychotic episodes’” (7). Burstow and LeFrancois, however, offer a more porous political affiliation based on notions of class praxis: they argue that it is ultimately a mistake for psychiatric survivors to “to keep people who do not share that oppressed identity at bay”; “identity politics alone will not win this fight – any more than the fight against classism would be won if we understood socialism as something that should only be theorized by and fought for by low-wage earners” (2014, 5).

Such competing models lead to continual debate within the movement about the appropriate degree of exclusion, inclusion, or alliance between alternative approaches. Consider, for instance, the boundary (if there is one) between the mad movement and the disability movement. Withers argues that the mad movement must give up “the conscious distancing of psychiatrized people from disabled communities” (2014, 118) apparent, for instance, in warnings against the “disabling” effects of psychiatric treatment – a tactic in which “[d]isability […] is used as shorthand to represent things that are bad, negative, and undesirable” (117). Once the mad movement has rid itself of disablism then it should, Withers believes, move toward “a careful, thoughtful, and respectful merger under the rubric of disability” (126). Beresford, however, while acknowledging a common experience of external definition and oppression (2000, 169-170), promotes a vision of *ad hoc* co-operation, rather than “trying to incorporate survivors in one monolithic movement” (171). An important sticking point is that “[w]hile some disabled writers […] include psychiatric system survivors in the social model of disability, […] some survivors reject this because they do not see themselves as having any kind of impairment” (170). One might note, for instance, that Farber’s spiritualized madness exemplifies this distinction, since it argues for “a distinctive mad sensibility different from that of ‘normal’ persons”; this spiritual sensibility “is an asset, not a defect”, and “a basis for ‘Mad Pride’” (2012, 9).

Hornstein certainly shares the broad aims of the mad movement: she has a fundamentally depathologizing view of madness, particularly voice-hearing, with a corresponding emphasis on psychosocial causation and context. Certain ideological co-ordinates can also be used to locate Hornstein more specifically within the complex field of the mad movement. Her overall project is very much an act of cultural assertion like that noted by Schrader et al. who observe how “[m]ad-identified groups have organized parades and rallies that, like the LGBTQ pride events after which they have often been modeled, function as transgressive, but also productive, displays of difference” (2013, 62). Schrader et al. argue that the Western culture of madness should now be part of clinical cultural competence, alongside, for instance, knowledge of non-Western cultural difference: they advocate an “appreciation of client values associated with a mad identity,” including “a basic understanding of the history and heterogeneity of the broader c/s/x [consumer/ survivor /ex-patient], mental diversity, and mad pride movements” (63). Hornstein clearly aims to draw upon the energies of this culturally affirmative strand of the mad movement in order to articulate an historical and cultural context within which madness is difference rather than deficiency. To this extent, her project somewhat resembles the indigenist strand of madness delineated by Louise Tam, who proposes to “fight for the ongoing cultural survival of Indigenous spirituality and healing” (2013, 297) – albeit with the qualification that *Agnes’s Jacket* is a work of cultural (re)construction more than preservation. It should also be noted (as will be discussed further below) that Hornstein, despite her investment in cultural difference, offers a porous vision of madness identity akin to that found in the political-revolutionary model promoted by Burstow and LeFrancois, who “theorize resistance against psychiatry as […] something that demands the attention of all who are critical” (2014, 5) and not merely psychiatric survivors in the narrow sense.

However, in order to appreciate Hornstein’s discursive intervention in the wider context of the mad movement (and also of the medical humanities), it is helpful to step back from particular, competing models of mad identity and to heed instead her use of a recurrent trope within the movement’s rhetoric: namely, the frequently expressed aim to “de-colonize” the experience of the mad and the consequent danger of discourses that replicate the psychiatric “colonization” of madness. This is a familiar, taken-for-granted rhetorical figure in mad studies. When Beresford, for instance, demurs from the incorporation of the mad into the disability movement, he warns against “the unintentional colonisation of survivors by the disabled people’s movement” (2000, 170). And when Simon McCarthy-Jones discusses the voice-hearing movement, he characterizes the Hearing Voices Network as part of “postmodernist, post-colonialist discourses” (2012, 95). His usage cites a *locus classicus* for the trope, namely the foundational work of Arthur W. Frank on contemporary illness narrative: “Applying Frank’s concepts, we can see that […] the modern experience of voice-hearing had been dominated by the technical expertise of psychiatry. This had led to psychiatry becoming the ‘spokesperson’ for voice-hearing, taking it over ‘just as political and economic colonialism took over geographic areas’” (91). The colonial metaphor is authorized by Frank in his influential *The Wounded Storyteller*, first published in 1995: “[j]ust as political and economic colonialism took over geographic areas, modernist medicine claimed the body of its patient as its territory, at least for the duration of the treatment” (2013, 10). This “colonization” brought the benefit of increased technological mastery over disease, but patients were dispossessed of their illness experience, particularly those with chronic illness. Now, in post-modern society, “[t]he post-colonial ill person, living with illness for the long term, wants her own suffering recognized in its individual particularity” (11). The vocabulary of “post-colonial” self-narration is valid, in Frank’s view, because “[p]ost-colonialism in its most generalized form is the demand to speak rather than being spoken for and to represent oneself rather than being represented or, in the worst cases, rather than being effaced entirely” (13).

Hornstein’s rhetoric elaborates this postcolonial figure, and its many variations, throughout the narrative of *Agnes’s Jacket*, according to her particular view of mad identity. Hornstein builds upon the potential of the “de-colonizing” trope by representing the developing mad community (including voice hearers) as the awakening of a transnational diaspora from its colonial dormission under psychiatry. She offers a transnational affiliation to the “de-colonized” mad who are modelled as a diasporic community of psychiatric survivors, voice hearers, and others commonly regarded as mentally ill. She thus recites a particular collective historical narrative to the mad, a story that goes beyond individual life narratives and binds them into a larger whole. In so doing, she continues and embroiders the provisional collective narratives of madness already found *in nuce* in identities such as “voice-hearer,” a category which, as Angela Woods explains, has “emerged as a culturally meaningful and politically charged identity enacted through a specific set of narrative practices” (2013, 263) – as Woods shows, individual story-telling by voice hearers draws upon shared narrative templates such as the “foundation myth” of the Dutch voice hearer Patsy Hague, a story that is “told and retold in multiple contexts and on multiple occasions” (264). However, Hornstein’s vision of the mad community goes beyond merely narrative resources and into the realm of material culture: as will be shown, her celebration of Agnes’s jacket, a textual and textile object, introduces this artefact, and the Prinzhorn collection, as a set of material objects apt for ethnosymbolic veneration.

It should be stressed that Hornstein’s intervention in mad identity is primarily implicit and practical rather than reflectively theorized. A commentator such as Rachel Gorman might focus analytically on the “hazards and promises of ‘Mad Nationalism,’” including the “danger that Mad identity […] will be absorbed into white, middle-class narratives of disability” (2013, 269), but Hornstein intervenes in mad identity in a much less explicit way. Key problems specific to Hornstein’s construction of the mad as a diasporic people will be considered in the critical discussion of her claims. Firstly, though, it is necessary to reflectively articulate Hornstein’s particular brand of mad nationalism. This means applying a literary attentiveness to *Agnes’s Jacket* in order to read closely its richly embroidered rhetoric of madness awakening from colonial dormission under the psychiatric empire, and to articulate the various overlapping paradigms for transnational identity that Hornstein consequently offers, from affirmative cultural difference, to ethno-symbolic nationalism, to literal and figurative diaspora.

## Hornstein’s ethnographic narrative

In order to intervene effectively in mad identity, Hornstein has to resolve, at least on a rhetorical level, a pressing difficulty: how can she extend, and transform, her authority as a clinical psychologist in order to speak credibly to a new identity for the mad? Part of her solution (or attempted solution, at least) is to mobilize and elaborate upon spatial metaphors of psychological interiority and to weave them into a vision of the quasi-national community of the mad, a movement awakening from its suppression under the totalitarian empire of psychiatry.

Hornstein, using her authority as a clinical psychologist, criticizes biomedical psychiatry from a familiar reference point, namely the methodological distinction between “explanation” and “understanding.” Biological psychiatry seeks to explain mental illness by subsuming phenomena under laws of cause and effect, such as those of brain functioning. Humanistic psychology and psychotherapy, however, try to make sense of the words and actions of the mentally ill, including phenomena such as voice-hearing or disorganized speech that might have been dismissed as merely the nonsensical epiphenomena of a disordered brain. This distinction between the methods of the natural sciences and those of the human sciences is familiar from the theory and practice of Karl Jaspers and R.D. Laing (Jaspers 1963; Laing 1965; Miller 2008; Hoerl 2013), as well as Frieda Fromm-Reichmann, as Hornstein herself has shown (2000, 122-172). Like earlier exponents of understanding such as Bert Kaplan (1964), Hornstein habitually glosses the communicative methodology with a spatial metaphor of interiority: “it must be possible to enter someone else’s experience and make sense of actions that from the outside might look inexplicable” (2012, xiv).

The familiar metaphor of the “inner world” is, however, elaborated by Hornstein in a novel way adapted to her intervention in identity politics. Hornstein states: “*Madness is more code than chemistry*. If we want to understand it, we need translators – native speakers, not just brain scans” (xix). The search for “native speakers” of madness introduces an ethnographic narrative whereby *Agnes’s Jacket* is an investigation of the mad as a different people with their own way of life and language in a literally or figuratively far-off place. Although Hornstein is literally a traveller to and around the UK, her journeys are rhetorically a passage to a “new world”: “During the semester, I’m a psychology professor; as soon as school ends, I’m back in the world of HVN [Hearing Voices Network]. They’re so radically different – in style, assumptions, and structure – that I feel as if I’m traveling a lot farther away than just to England” (44). The metaphor is carefully woven into the detail of the text: for instance, as part of her exploration of this “intriguing and disturbing new world” (22), Hornstein finds herself on a London bus that “stops outside a cavernous pub called the World’s End” (23). Hornstein accordingly models the Voice Hearers and other psychiatric survivors to whom she speaks as ethnographic informants: these “interpreters, people whose understanding of madness comes from firsthand experience” have been to her “like native guides” (xiv). Her numerous interviews with such people throughout the book are presented not as clinical interviews but rather as ethnographic fieldwork (albeit – as will be shown below – of a somewhat old-fashioned variety with little investment in ideals of shared authority). The paradigm established is familiar from the classic anthropological work of Bronislaw Malinowski and others in which “[t]he field-worker is ‘adopted’, ‘learns’ the culture and the language”, and undertakes “a sort of mini-immigration” (Clifford 1992, 99). This rhetoric within *Agnes’s Jacket* primes the reader to accept that madness is the way of life of a people or group who occupy a distinct “world” and possess their own culture and language. Moreover, the reader is encouraged to accept that Hornstein, by virtue of her “mini-immigration,” is a credible inside guide to this emerging “new world.”

In synergy with this cultural model of madness, Hornstein offers an overlapping de-colonizing rhetoric of suppressed or latent nationhood. She is sensitive to the ethno-symbolic materials of collective identity, such as those analysed by Anthony D. Smith, for whom “the cultural elements of symbol, myth, memory, value, ritual and tradition” are “crucial to an analysis of ethnicity, nations and nationalisms” (2009, 25). When Hornstein alludes, for instance, to a tradition of activist literature in *Agnes’s Jacket*, she makes a somewhat contentious claim that many of these texts have been intergenerationally transmitted by the mad: “Patients have always recognized the power of these classic texts, passing them from generation to generation (often as contraband on locked wards)” (204). Whatever the historiographic merits of this claim, Hornstein’s implication is that these texts are analogous to the cultural patrimony of a suppressed national tradition handed down from generation to generation. Indeed, this textual corpus allows a further deployment of territorial and national imagery when Hornstein metaphorises her bibliography of madness narratives as a list of texts smuggled out of a closed country:

Communiqués from the world of madness are far more numerous than one might think. Despite every attempt to silence them, hundreds of patients have managed to get their stories out, at least in disguised form. More than six hundred first-person narratives of madness have been published in English alone, the earliest in 1436, the most recent, last month. (xii)

The paradigm here is borrowed from the popular understanding of political samizdat texts such as Aleksandr Solzhenitsyn’s *GULag Archipelago* (1973), which fostered the conception of “samizdat as the forum of ‘heroic and uncompromising’ truth wielded by dissident-warriors struggling valiantly against the totalitarian regime to bring about its eventual demise” (Komaromi 2004, 599). Hornstein’s introductory citation of Timothy Garton Ash’s *We the People* (1990), a journalistic account of the demise of the Soviet bloc, provides a parallel generic cue for *Agnes’s Jacket* itself (xxv). Hornstein’s text is, according to its own rhetoric, a piece of reportage on the nation(s) emerging from the dying days of the psychiatric empire.

There are further dimensions to Hornstein’s offer of nationhood in her depiction and promotion of quasi-religious practices within the mad community. As Smith reminds us, “nationalism may be secular; but, seen as a set of reiterated cultural practices, it appears in a new guise, as a form of religion, one that is of this world and human centred, certainly, and thus secular, but a religion nonetheless” (2009, 76). This analytic framework helps to explain Hornstein’s weaving of ritual motifs into her account of the Freedom Center, a group which for two years she attends “religiously” (60). Alongside commensal rituals such as the sharing of “ginger cookies” (57), she records “a reverence to people’s attentiveness to one another” apparent when one person speaks to the group, and “everyone else is suddenly silent, as if the opening chord of the church organ has just sounded” (57). Building upon this representation of spontaneous religiosity, Hornstein positions Agnes’s jacket as a sacred national relic. The holiness of Agnes’s jacket – as perceived by Hornstein – is particularly clear in a private viewing at the Prinzhorn collection: “The front of the case swings open and I step back involuntarily, awed by the jacket’s silent power” (247): “The physical presence of the garment […] has the totemic power of a relic or a shaman’s robe. […] we’ve been transported to a sacred space, with the jacket the focus of our reverent observation” (248). Readers (mad or otherwise) are invited to recognize the jacket as a sacred relic, something to be commemorated in collective ritual. Potential objections to this sacralising invitation are indirectly disarmed when Hornstein later asserts that the Rosetta Stone – an important metonymy for translation and thus of challenge to the supposedly “ununderstandable” – is itself revered by casual visitors to the British Museum: “for millions of people, seeing the Rosetta stone is like encountering a holy relic. Since neither the stone itself nor the content of its message makes it important, what people revere is its decipherment and the recovery of ancient Egyptian history that this made possible” (266). The prevalence of reverent feelings among visitors to the British Museum is not actually ascertained, but Hornstein’s generalization of her own response helps to legitimate her veneration of Agnes’s jacket and her implicit transformation of the Prinzhorn Collection into a vast reliquary.

The rhetoric in *Agnes’s Jacket* builds upon metaphors of psychological interiority in order to authorize Hornstein’s construction of the mad as existing in a separate “world” with a distinct culture that can be ethnographically recorded. By offering the break-up of the USSR as an analogy, Hornstein constructs the mad movement as a nation awakening from its colonial dormission under the psychiatric regime. She consequently offers materials of ethno-symbolic nationhood in: the equivalent of a national literary tradition; ritual practices for the secular religion of nationhood; and artefacts that can function as a focus for sacred rituals of remembrance. Moreover, a notable transformation occurs in Hornstein’s model of mad identity. As madness is “de-colonized,” and liberated from psychiatry, it becomes – at least in Hornstein’s vision – a somewhat porous identity, potentially open to those who have no psychological claim to mad experience. If one accepts Hornstein’s rhetoric, then, with sufficient dedication, one can undergo a “mini-immigration” into the community of the mad, without actually laying claim to psychiatric survivor status. The ethno-symbolic vision offered by Hornstein further mobilizes mad identity beyond a psychiatrized population: text, artefact, and ritual are as much elements of membership as any particular psychiatric or psychological criteria. Anyone, in principle, can revere Agnes’s jacket, just as – supposedly – millions of both domestic and international visitors to the British Museum spontaneously revere the Rosetta Stone.

## Diaspora

The complexities of Hornstein’s increasingly porous vision of mad identity will be discussed later. But even within the discursive logic of her text, there seems a clear obstacle to her rhetoric of awakened post-colonial ethno-symbolic nationhood. Unlike Sierra Leone, Belize, or Estonia, the mad movement has no concomitant territory, for it is of course very widely geographically dispersed within and across nation-state boundaries. Hornstein, however, turns this fact to her advantage by drawing upon the contemporary political and intellectual momentum found in discourses and practices of global, post-national community. In Hornstein’s most literal offer, the mad are a diaspora without an origin: rather than being formed by an original historical displacement from a territory, they are instead a scattered group coalescing into a single transnational consciousness. On a more metaphorical level, however, Hornstein simultaneously preserves familiar diasporic elements in the form of a psychologized territory: there is a homeland, of sorts, namely an “inner” psychic geography liberated from the colonial depredations of psychiatry.

There can be little doubt about the contemporary allure of transnational discourses. Steven Vertovec outlines our contemporary fascination with transnationalism as “a condition in which, despite great distances and notwithstanding the presence of international borders […], certain kinds of relationships have been globally intensified and now take place paradoxically in a planet-spanning yet common – however virtual – arena of activity” (1999, 447). Relationships within but crucially also across national boundaries lead to new forms of social networks, personal and collective identity, cultural activity, economic relationships, and political engagement, alongside a transformed sense of localism (449-456). Hornstein’s rhetoric is prudently selected, for in presenting the mad as an awakening diasporic community, she borrows a strategy already used successfully with other minority identities such as deafness and homosexuality. Harlan Lane, for instance, declares unequivocally that “the sign language-using minority in the United States, the Deaf-World, is best viewed as an ethnic group” rather than “as a disability group” (2005, 306). The idea of a diaspora without origins, one that coalesces rather than scatters, is also found in the model of a gay diaspora – as Alan Sinfield remarks, “Instead of dispersing, we assemble” (1996, 280).

In Hornstein’s historical analysis, similar processes of identity formation are crucially enabled by the large-scale transferal of mental health care in the West from hospital to community:

*de-institutionalization* created the structural conditions for mental patients to collaborate. For the first time in history, people who had been institutionalized in different hospitals, in different parts of the country, or even in different countries could come together, discuss their individual experiences, learn from one another, and put forward their own ideas about how the mind works. (165)

This “de-colonizing” of the voice of the mad, initiated by de-institutionalization, is continued in Hornstein’s narrative by important congregations within the movement such as when “[i]n October 1987, the World Conference on Voice Hearing is held in Utrecht. For the first time in history, 250 people who hear voices meet together” (32). Naturally, Hornstein does not propose to form a future literal homeland for the madness diaspora, but as Robin Cohen explains, there are indeed diasporic communities, such as the Afro-Caribbean diaspora, which are wholly “deterritorialized” – “having lost their conventional territorial reference points,” they “have become in effect mobile and multi-located cultures” (2008, 124).

Nonetheless, alongside the image of a deterritorialized diaspora coalescing into a group identity, there is in *Agnes’s Jacket* a more figurative reworking of familiar diasporic elements such as victimhood and even homeland. Cohen reminds us that the Jewish prototype of “the forcible dispersal of a people and their subsequent unhappiness, or supposed unhappiness, in their countries of exile” (2008, 35) exemplifies a larger class of “victim diasporas” distinguished by “the idea of dispersal following a traumatic event in the homeland, to two or more foreign destinations” (2). The emphasis in Cohen’s ideal type on “a traumatic event in the homeland” illuminates an extra significance in Hornstein’s frequent espousal of traumatic aetiology. Hornstein argues for trauma as a cause of voice hearing: “80 to 90 percent of people who hear voices – whether psychiatric patients or not – link traumatic events to the origin of their voice hearing” (40). More generally, she claims that “if we are to understand madness” then “[w]e have to go back to the language of trauma,” of “demonstrable abuse that occurred in the family or in psychiatric institutions themselves” (xxi). Although psychosocial causation is a familiar tenet of the mad movement, Hornstein’s use of the word “trauma” also mobilises certain historical and political connotations, for the concept of psychological trauma has in recent years wandered out into the world of historiography and entered into a wider “memory discourse” in which “even the most rigorous scholar is free to speak of the memory of events that happened hundreds of years distant or to speak of the memory of an ethnic, religious, or racial group” (Klein 2000, 136). In certain psychoanalytic models of collective memory, repressed emotions of suffering and guilt are handed down from generation to generation, so that each is haunted by “unresolved, phantomlike residue[s] of the past acquired through often unconscious, transferential processes of identification with loved ones and their encrypted experiences” (Lacapra 1998, 64). While the manifest aim in Hornstein’s trauma theory is to present a properly psychological rather than neurological cause for madness, the statement also implies, in terms of diaspora and memory discourse, that the mad are united by shared trauma. The trauma in question is not a discrete historical event but rather an indefinite number of personal traumas that nonetheless are – in Hornstein’s view – sufficiently similar to legitimise a sense of collective victimhood and displacement: the mad are a diasporic community of metaphorical refugees from traumatic situations.

Hornstein also discovers (or invents) a figurative spatial homeland for these displaced persons by reviving a metaphor that was particularly favoured in post-war counterculture: that of an unexplored “inner space” of extraordinary states of consciousness. A concise, early expression may be found in Aldous Huxley’s introduction to his experiments with mescaline in *Heaven and Hell*:

A man consists of what I may call an Old World of personal consciousness and, beyond a dividing sea, a series of New Worlds – the not too distant Virginias and Carolinas of the personal unconscious and the vegetative soul; the Far West of the collective unconscious, with its flora of symbols, its tribes of aboriginal archetypes; and, across another, vaster ocean, at the antipodes of everyday consciousness, the world of Visionary Experience. (1956, 10)

In *Agnes’s Jacket*, the content of this spatial metaphor of consciousness as terrain varies enormously, but the general form persists in various texts and informants cited by Hornstein. We find phrases such as “the icebergs of deep consciousness” (179), the “vast and bottomless caverns of naked madness” (163), or the experience of madness as being “*inside a labyrinth*” (210). Even everyday affective life can end up in alien territory: Hornstein discusses a woman raised in a stifling family context wherein “*emotions ended up on the other side of no-man’s-land*” (148). The discourse is most clearly authorized when Hornstein cites the Prinzhorn curator, Inge Jadi: “The [Prinzhorn] Collection is a provocation. It points beyond the limits of so-called reality to the infinitely vast landscape of inner truths which mocks any claim to absolute power over our normal world” (240). The inner space of extraordinary states of mind is thus a figurative homeland for the mad, one which they may hope to re-occupy by overcoming the meanings imposed upon it by biomedical psychiatry. As Hornstein says, “For survivors to make sense of what has happened to them, they must ‘decolonize’ their experiences and create narratives framed outside the medical model of their doctors” (160).

Although it elaborates the pivotal discourse of “de-colonization,” Hornstein’s diasporic offer may seem to sit uneasily with her implicitly porous ethnosymbolic rhetoric. Nonetheless, as Rogers Brubaker notes, there is “a tension in the literature [on diaspora] between *boundary-maintenance* and *boundary-erosion*” (2005, 6). Admittedly, the former may seem “an indispensable criterion of diaspora,” since it “enables one to speak of a diaspora as a distinctive ‘community’, held together by a distinctive, active solidarity” (6). However, “a strong counter-current emphasizes hybridity, fluidity, creolization and syncretism” (6). This latter intellectual formation, as Stéphane Dufoix notes, is represented by postmodern theorists such as “Stuart Hall, James Clifford, and Paul Gilroy” (2008, 24) and provides “a decentralized vision more focused on the frontiers of the diaspora than its core” (25). In light of this counter-formation, Brubaker argues, “we should think of diaspora not in substantialist terms as a bounded entity, but rather as an idiom, a stance, a claim” (2005, 12): the term “diaspora” is first and foremost “a category of practice” rather “a category of analysis” and as such “is used to make claims, to articulate projects, to formulate expectations, to mobilize energies, to appeal to loyalties” (12). Viewed in this light, then we can understand Hornstein’s discourse of diaspora less as a scholarly analysis and more as an active intervention in identity formation mounted from the periphery of the survivor movement. The implicit term “diaspora” in Hornstein’s work is therefore a practitioner category; it is part of her marketing of new ethno-symbolic materials for mad (trans)nationalism, rather than a strictly analytic sociological category.

## Critical discussion

The dominant identitarian thrust of *Agnes’s Jacket* is that the mad are, or should be, consciously united as a “de-colonized” (trans)national diasporic community cleaving to a way of life worthy of recognition and respect. However, there are a number of challenges that one might pose to this mobilization of identity politics such as: its Western-centred investment in identity politics; the limits of depathologisation though assertion of identity; the potentially oppressive stereotypes of new mad identities; and the risk that Hornstein herself may “re-colonize” madness.

The parochialism of Hornstein’s model is clearly problematic. To call for recognition of diasporic identity is no doubt significant within Western high income countries, particularly in North America. As Sinfield notes, the idea of gay ethnicity has been attractive in the US “in the period when ethnicity, following the precedent of the Black Civil Rights movement, has offered the dominant paradigm for political advancement” (1996, 271). North America, and to a lesser extent the UK, have endorsed the politics of identity, to the extent indeed of largely displacing the politics of redistribution in mainstream political discourse (Michaels 2006). But what relevance do such strategies have for low to middle income countries (LMICs)? There are already widely noted concerns about the exportation of Western biomedical expertise to LMICs as part of the so-called “scaling up” of services. Suman Fernando, for instance, warns that the development of services on a Western model may erode “indigenous, culturally acceptable means of help and support for people with personal or social problems resulting in distress” (2014, 128). To export Hornstein’s identitarian advocacy could be simply to impose an extra layer of (literal) neo-colonialism – a Western response to the West’s own problems with biomedical psychiatry, one that overlooks resources in LMICs for dealing with severe mental illness. Juli H. McGruder, for instance, has shown how for families in Zanzibar with schizophrenic members there is value in “the belief that all adversity is sent from Allah for a purpose one cannot know, and that preternatural spirits are active in producing deranged behaviour” (2004, 278). This belief, set in a context of more reserved emotional expression, “exonerates patient and family, and sustains tolerance and acceptance of the patient” (278). Such indigenous, non-Western discourses and practices have not, or at least not yet, been “colonized” by the expert, professional knowledge of biomedical psychiatry.

Even within the discourses and practices of Western identity politics, there are difficulties for the model of madness as identity. Hornstein’s depathologizing strategy may be understood using the conceptual distinction between “parameters” and “limits”: “Some of our circumstances […] act as *parameters* […] defining what it is for us to have lived a successful life”; “Others are *limits* – obstacles that get in the way of our making the ideal life that the parameters help define” (Appiah 2005, 111). Appiah explains the “reversible-raincoat nature of these terms”: “for many deaf people deafness is not a limit but a parameter: they are not trying to overcome a disability; they are trying to live successful lives as the hard-of-hearing people that they are. A condition becomes an identity – the deaf become the Deaf” (112). Indeed, one of Appiah’s examples of the limit-parameter gestalt switch is homosexuality (111-112), which has migrated over the last few decades from a category in psychiatric nosology to a highly visible and assertive identity. Just as the deaf have become the Deaf or homosexuals have become the gay community, so for Hornstein the mentally ill may become the mad (without an upper-case M in her usage). Thanks to the efforts of HVN and its allies (including Hornstein), voice hearing seems to be undergoing such a transformation – from first-rank psychotic symptom to a mode of human diversity that is no more inherently pathological than non-heteronormative gender and sexuality. Furthermore, there is even a strategy of reversal within the wider mad movement, whereby psychological normality is the limiting circumstance. Farber’s contemporary discourse of “mad gifts” continues the 1960s anti-psychiatric suspicion of everyday normality: madness is an “asset,” a special “sensibility” that can “promote spiritual growth,” whereas “adjustment to society” is actually pathological, since society itself is “insane” (2012, 9).

As the example of Farber suggests, such mobilization of identity may be highly contested: the same limit-parameter switch is attempted by the pro-ana [pro-anorexic] movement, particularly in its online interaction (indeed, the title of a now-defunct leading pro-ana website, “Anorexic Nation” [Ferreday 2003, 283-284], clearly employed the rhetoric of a deterritorialized diaspora). Although the agenda of pro-ana websites is heterogeneous, there is nonetheless a clear strand of activity that defines anorexia as a way of life rather than as a mental illness (Roberts Strife and Rickard 2011). Such advocacy is obviously contentious and shows the potential difficulties in a broader programme of madness as identity. The attempted creation of new parameters of identity may motivate a variety of responses, from whole-hearted endorsement to caution or hostility, whether from the general public or from within the proposed identity group. Indeed, one could well imagine a former anorexic who might have deep reservations about the psychiatric understanding of anorexia but without thereby endorsing the notion of anorexia as parameter of a successful life.

Even granting a successful public transformation from limit to parameter, a new identity born of former psychopathology will face the same issues as any collective identity, such as the extent to which “normative stereotypes” (Appiah 2005, 195) may be open to internal diversity and contestation, or how far one may opt out of the ascription of a particular identity. Voice-hearing or madness as identity might well be unfair to marginal groupings (how accommodating would the future identity be to residual “self-hating” voice hearers?), oppressive to those for whom their voice-hearing has no particular significance (the “lapsed” or “secular” voice-hearer, as it were), or exclusive of those unsympathetic to Hornstein’s various shibboleths (for instance, a voice-hearer who has no reverent feelings for Agnes’s jacket). The problem is familiar from other account of minority identity: writing with respect to homosexuality, Sinfeld has for instance been far more cautious about the “ethnicity-and-rights” model (1996, 272), drawing attention to, amongst other problems, the risk of a homogenizing model of gay identity (288-289).

Moreover, a further issue can be discerned in Hornstein’s text: the risk that Hornstein’s discourse itself “re-colonizes” the mad movement. While attending Freedom Center meetings, Hornstein receives a peculiar methodological caution: “Will had warned: ‘You’d better not be a tourist. We don’t want any anthropologists, either. We’re not a bunch of natives, letting you visit our indigenous community. Freedom Center isn’t a field site for participant observation.’ I’d agreed with him” (54). Hornstein’s agreement marks an important unspoken implication whereby she positions herself as an inside member of the survivor movement rather than as an anthropological participant-observer. To validate this claim to membership, Hornstein recounts her psychological benefits from attendance at Freedom Center meetings: “I’ve shown up at one of these meetings filled with worry about some personal difficulty or frustrated with my colleagues or mired in impotent despair […]. As I listen to people talk about trauma far more serious than anything I’ve experienced […], I marvel at the transformation of my own feelings” (61). This recurring “transformation” marks, in essence, a successful narrative of conversion, with a key turning point being Hornstein’s first Freedom Center meeting, where her unguarded disclosure of doubts about biomedical psychiatry – “I’m startled by the words that are tumbling out of my mouth; I’d never intended to be this revealing” – marks a transition from participant observation to authentic membership: “I feel as if I’ve just passed some test, one harder than I was anticipating” (55). Indeed, even without such a conversion narrative, the wider ambition of *Agnes’s Jacket* testifies to Hornstein’s claim of intellectual and cultural leadership from within the community of the mad. Hornstein, after all, crafts various overlapping offers of identity from the cultural materials available, including the sacred relic provided by Agnes’s jacket to which she herself responds with a sincerely felt awe and reverence that she invites her readers (mad or not) to share.

Hornstein therefore claims to have become a member of the mad community – and a significant one – yet to have done so without (as far as one can tell from the text) any particular claim on her part to the status of psychiatric survivor. Moreover, Hornstein’s conversion would seem open to a correspondingly wide spectrum of her readership: as the reader’s *alter-ego* on a journey through the world of the mad, she is a proxy to those who are not psychiatric survivors, voice hearers, or mentally ill in any recognized clinical sense, but who might share, for instance, her reverence of artefacts such as Agnes’s jacket, or her doubts about psychiatry. This may seem an entirely innocuous invitation. The Janus-faced character of diaspora discourse, both maintaining and eroding boundaries, would seem to indicate nothing improper – in principle, at least – about extending identities such as “mad,” “psychiatric survivor,” or “voice hearer.” That the boundaries of any particular identity are flexible and in process is hardly unusual: as Richard Jenkins observes, “collective identification and its boundaries are […] generated in transaction and interaction and are, at least potentially, *flexible*, *situational* and *negotiable*” (2014, 133).

However, notwithstanding the potential for a more porous community of the mad, there would appear to be a significant ethical and methodological problem in Hornstein’s self-ascribed “insider” position. The difficulty lies in the conceptual model to which she subscribes, that of metaphorical “decolonization” via self-narration: “For survivors to make sense of what has happened to them, they must ‘decolonize’ their experiences and create narratives framed outside the medical model of their doctors” (160). As indicated above, this is a key metaphor widely endorsed in research conducted with the mad community. For Simon McCarthy-Jones, for instance, the diverse experience of voice-hearing was colonized by modern psychiatry – a political analogy which, as shown above, he consciously draws from the foundational work of Frank (2012, 91). Jacqui Dillon and Rufus May provide an influential statement of this “de-colonizing” model for research methodology with the mad:

Clinical language has colonized experiences of distress and alienation. Consequently, many accounts of recovery seem to be about a decolonizing process, a reclaiming of experience. Through this sharing of experience we are told new stories – counter-narratives which offer diverse representations of survival in adversity. These cultural documents inform us that recovery is possible, that our experiences are meaningful and that each person’s experience is unique. (2002, 25)

In light of this methodological commitment, the mad are recognized as possessing an expertise by experience that complements, but also challenges, the professional expertise of academics and clinicians – Angela Sweeney, for instance, advocates a model in which “service user knowledge” is “based on experience” (2009, 32). As Peter Beresford explains, academic researchers who acknowledge expertise by experience deprecate the “‘objectivist’ approach to research” in which “if an individual has direct experience of problems like disability or poverty, or of oppression and discrimination, […] what they say will be seen as having less legitimacy” (2013, 146). The positive alternative to such (implicitly “colonial”) research is a model of “co-production” of knowledge between academia and the mad:

User or survivor controlled research makes it possible to develop a counter discourse to psychiatry, based on survivor knowledge and “lived experience” that is evidence-based and which can challenge the “scientism” of psychiatry, where mental health service users shape the research question and focus, are involved in carrying out the research, producing its findings, disseminating them, and deciding on follow-up action and are in control of all these aspects of the research process. (Beresford and Menzies 2014, 91)

In this paradigm, the academic researcher plays a more facilitative and dialogic role. For instance, Marius Romme and his co-editors elicit and edit the testimony of fifty voice hearers in their collection, *Living With Voices* (Romme et al. 2009). After an initial request for spontaneous narratives, the editors made an offer to work together with participants on interviews, which were subsequently edited “to a maximum of four A4 typed pages, focusing on what was said about recovery, but keeping the words used by the voice hearer in the interview […] in order to remain as authentic as possible to the way people have expressed themselves” (Romme and Morris 2009, 5). The ideal of decolonized experience is, for instance, explicit in some of the testimony offered by contributing voice hearers. Eleanor Longden states her aspiration to be “part of this movement to change the way that we relate to human experience and diversity,” and concludes with the apothegm, “Your soul can’t breathe when your mind has been colonised” (Romme et al. 2009, 146).

The “decolonizing” ideal of knowledge co-production can also be expressed in the terminology of “shared/sharing ownership,” a principle well exemplified by the collaborative work of the oral historian Megan Davies and the psychiatric survivor Lanny Beckman. Their collaborative chapter on the origins of the Mental Patients Association in 1970s Canada is constructed as dialogue alternating between Davies (the professional historian) and Beckman (the nominal interviewee), with their contrasting perspectives on this joint enterprise:

*Megan*: I am a social historian of British Columbia health practices. […] I was looking for stories of the Mental Patients Association (MPA), a radical self-help group that formed in Vancouver during the turbulent years of the early 1970s.

*Lanny*: I was leading a quiet life as a semi-recluse when this Megan J. Davies person banged down my front door. She said she wanted to interview me about an organization called the Mental Patients Association, which she claimed I founded in 1970. She said I had boxes of early MPA documents in my basement – how she knew this I have no idea, but she was right. (Beckman and Davies 2013, 49-50)

As part of this co-production, there is a degree of honesty about their differing backgrounds. Davies admits that “[a]s project historian, I was willing to revisit the way in which I use theory, analysis, and synthesis, but not to abandon these elements of my craft,” whereas Beckman is troubled by “a common practice and failing in academia, […], to use jargon to render intelligible ideas difficult to follow” (56). The resultant book chapter, in content and form, as well as underlying process, expresses a significant commitment to the “co-production” of knowledge and the consequent synergies and tensions.

There are, no doubt, problems inherent in the principle of co-production and shared authority. Academics may be tempted to proceed, for instance, as if “authority were some dammed up reserve ‘we’ release so that it can flow down to ‘them’, generating in the process power for transformation at and from the bottom” (Frisch 2003, 112). It may also prove difficult for “researchers engaged in collaborative projects to draw conclusions that might prove unpopular with community partners” (High 2009, 20). Nonetheless, on methodological grounds alone, Hornstein owes in *Agnes’s Jacket* a fuller account of her research process and its limitations. Indeed, when considered in light of co-production and shared ownership, the porosity of mad identity in Hornstein’s work may seem merely a way of concealing the ongoing “colonial” dimensions of her project. Although Hornstein is open about her personal motivations and frequently reproduces lengthy direct quotations from her informants, her broader commitment to co-production and shared ownership is unclear and perhaps even seriously deficient. The testimony of the mad appears but without the transparency about process offered by a volume such as *Living with Voices*, where the testimony of each participant is at least given substantial independent weighting. Nor is there the same even-handed dialogic approach as found in the work of Davies and Beckman; we don’t even know, for instance, if “Will” at the Freedom Center felt his admonitions had been heeded by his academic guest. Hornstein’s informants thus seem quite unlike collaborators in the mould described by Beresford and Menzies (above), where they would take an active role in shaping the questions, carrying out the research, producing and disseminating the results, and working out the practical inferences. Viewed in this light, Hornstein’s depiction of her supposed transformation through various rites-of-passage to authentic membership of the mad community is a way of disarming objections to her deficient methodology. If we, her readers, can be beguiled into accepting Hornstein’s inclusion in the community about which she writes, then we will be inclined to view her as someone authentically reclaiming the colonized experience of the mad. Her ethno-symbolic (trans)national rhetoric facilitates this artful misdirection precisely by detaching madness from its grounding in first-person experience of psychiatric power imposed upon personal distress. Even the choice of Agnes Richter as a central figure may invite suspicion: why choose a nineteenth-century psychiatric patient whose inner life is lost to history when they are so many living members of the mad community who are available to enter into a real dialogue about their experiences?

Nonetheless, while this methodological critique of Hornstein is valid, the inference that her research “colonizes” the mad community is perhaps only a partial truth. Even within the parameters of the (post-)colonial metaphor, there is a qualified legitimacy in Hornstein’s self-identification as “mad.” We might deprecate her particular narrative of community membership and wonder at the proposed equivalence between, say, a psychiatric survivor and someone who thinks Agnes’s jacket – or even just *Agnes’s Jacket –* is awesome. But, as Burstow and LeFrancois argue, we need not

artificially create dichotomies and divisions between activists and academics, between the openly psychiatrized and those may refuse classification of their experiences or those who have escaped psychiatrization. The point is, given that we are all at risk of psychiatrization, we cannot afford to exclude the work and theorizing of anyone engaging in radical and activist scholarship, if we are to succeed. (Burstow and LeFrancois 2014, 5)

Such is the “imperialism” of biomedical psychiatry; we are all potentially “colonized” by its technical expertise. Hornstein’s distress may seem small beer – “filled with worry about some personal difficulty or frustrated with my colleagues or mired in impotent despair” (61) – but psychiatry already has a potential territorial claim over worry (as anxiety) and despair (as depression). To refuse Hornstein her own act of resistance, as she reclaims her more quotidian experience, seems to neglect the expansionist proiect of the psychiatric empire.

Moreover, the metaphor of colonization versus decolonization – not only deployed by Hornstein but also potentially against her – requires closer consideration, despite its utility, and its promotion by distinguished commentators such as Frank. On a straightforward level, there is something potentially colonial within this model, for the valorization of psychiatric “decolonization” may occlude what is needed for intersectional groups who have already been colonized the literal, old-fashioned way. As Tam argues, “Theorizing psychiatric violence as being like racism or like colonialism writes out the existence and relevance of colonial gender violence in the foundations and ruling organization of mental health services” (2013, 297). So, for instance, the mad community in North America may appropriate “spiritual practices such as shamanism” but “without acknowledging the fight for the ongoing cultural survival of Indigenous spirituality” (297).

The metaphor of “decolonization” can also to some extent defend Hornstein’s ethno-symbolic vision of a transnational mad community, even if the argument is not one that she herself makes. Literal decolonization resulted in the emergence of post-colonial nations. Yet what these nations had most in common was the experience of decolonization, including resistance to discourses that constructed them as homogeneous racial and/or Oriental Others. Anthony Appiah repudiates, for instance, the colonial mythology of a unanimous African cultural spirit: “Whatever Africans share, we do not have a common traditional culture, common languages, a common religious or conceptual vocabulary” (1992, 41). To pursue the analogy further, why should one expect unanimity in discourse and identity when the mad are “decolonized”? The experience of psychiatric oppression in its various forms is presumably an important commonality, but there seems no reason to presume any further unanimity.

Indeed, as Dillon and May have indicated with respect to voice hearing,

the frame of reference within which the storying of experience takes place can be varied. Thus voice hearers may choose from a range of explanatory systems to narrate their experience. Examples are socio-political, spiritual and paranormal, as well as psychological and bio-medical frameworks. (2002, 26)

The identity politics of voice hearing are particularly complex. As Woods explains, there are “many different forms of voice-hearing” (2013, 267); this is a point reinforced by recent research by the Wellcome Trust-funded Hearing the Voice project (Hearing the Voice) showing the phenomenological diversity in self-identifying voice hearers with a sizable minority deeming “voice an inadequate term for their experience, instead using terms such as ‘intuitive knowing’ or ‘telepathic experience’, or descriptors such as ‘alters’, ‘parts’, or ‘fellow system members’” (Woods et al. 2015, 325). Moreover, the “people who hear voices all do not share the same experiences, values, beliefs and histories” (2013, 267): to identify as a voice-hearer is typically to “share not just a common experience of hearing voices but also a (frequently negative) experience of mental health services” (265). Thus, not all who identify as “voice hearers” hear voices, and not all who hear voices identify as “voice hearers.”

Viewed in light of such considerations, Hornstein’s inscription of herself into the mad movement is less ethically problematic than it may appear. As residual psychiatric categories are challenged, so the formerly “colonized” subjects may be able to identify in unpredictable ways as members of communities, existing or new, in which previously pathologized experiences (such as voice-hearing) need no longer furnish constitutive shibboleths. To take just one emergent example: why should the recipient of spiritual voices feel a particular affinity with other voice hearers, such as those who feel persecuted by their voices, or those who experience the voices of specific, life-historical individuals? Such an individual, if effectively “de-colonized,” might define herself as primarily a member of a religious community – one who happens to hear voices – rather than as a “voice hearer.” A plausible historical comparison can be found, for instance, in McCarthy-Jones’ discussion of nineteenth-century Anglo-American spiritualism. This movement offered “a very different discourse […] which assigned meaning and value to the content of voices” as a mode of religious subjectivity that was part of a whole range of spiritualist belief, practice, and experience (2012, 70). Indeed, the contemporary spiritualist movement although much smaller and far more marginalized than its nineteenth-century predecessor continues to offer meaning and a broader community that includes, but is not limited to, individuals (“mediums”) who have anomalous experiences, including visions and voice hearing (Roxburgh and Roe 2014).

## Conclusion

*Agnes’s Jacket* is a text significant for both its critical account of psychiatric models of madness and for its intervention in the identity politics of the mad movement. The latter has been the concern of this article, which has aimed to articulate and critique the model of mad identity offered to the mad movement (and beyond) by this culturally significant text. As my reading of *Agnes’s Jacket* shows, Hornstein offers a resignified vision of mad identity that embroiders the central trope of an “anti-colonial” struggle to reclaim the interior, experiential world colonized by psychiatry. A series of literal and figurative appeals makes recourse to the inner world and (corresponding) cultural world of the mad as well as to the ethno-symbolic cultural materials of dormant nationhood. This rhetoric is augmented by an appeal to the contemporary political energy of diasporic community. The mad are a diaspora without an origin, coalescing into a single transnational community; they are also persons displaced from a metaphorical homeland, the “inner” world “colonized” by the psychiatric regime.

Yet, while one might admire the rhetorical skill apparent in *Agnes’s Jacket*, there are problems in its model of mad identity that remain to be more fully explored, particularly from voices within the mad community. Hornstein’s “ethnicity-and-rights” response to the oppression of the mad is symptomatic of Western parochialism, while her proposed transmutation of putative psychopathology from limit upon identity to parameter of successful identity is open to indefinite *ad hoc* contestation – what is plausible with respect to voice-hearing may be less so, for instance, with respect to anorexia. Moreover, Hornstein’s self-ascribed insider status in relation to the mad community points toward a complex critical debate. This supposed standing, facilitated by her ethno-symbolic model of a mad diaspora, may be understood as a manoeuvre to disguise the betrayal of her own “de-colonizing” aspiration to recognize the experiential expertise of the mad: the degree of explicit knowledge co-production in *Agnes’s Jacket* is rather limited, but this deficiency is veiled by Hornstein’s apparent “insider” voice. However, in Hornstein’s defence, the “de-colonizing” logic of the mad movement points also toward a possible space for mad identities in which residual psychiatric categories may have much less significance. Her porous model of mad identity may presage a partial dissolution of psychological and/or psychiatric criteria for membership and also an unravelling of threads previously bound together by the essentialism of psychiatric categories. Hornstein’s text is an intervention as much as an analysis, and its plausibility will to some extent depend upon its eventual historical efficacy in the ongoing process of mad identities. The aim of this article has been to assist in making this process a more reflective one by critically articulating the discursive logic of *Agnes’s Jacket*, a text that is undoubtedly a significant cultural landmark for the mad movement.

## Endnotes

^1^ Following Hornstein’s own usage, I will employ the term “mad” to refer to this grouping, and will do so without scare quotes.
